# Feasibility and acceptability of FOotpaths foR adolescent MAternal mental HeAlth (FOR MAMA): A co-designed intervention for pregnant adolescents in Malawi

**DOI:** 10.1017/gmh.2024.76

**Published:** 2024-10-24

**Authors:** Wezi Mhango, Daniel Michelson, Darya Gaysina

**Affiliations:** 1 School of Psychology, University of Sussex, Brighton, UK; 2 Department of Psychology and Medical Humanities, University of Malawi, Zomba, Malawi; 3 Department of Child and Adolescent Psychiatry, Institute of Psychiatry, Psychology and Neuroscience, King’s College London, London, UK

**Keywords:** adolescence, mental health, psychosocial, intervention, LMICs

## Abstract

This study aimed to assess feasibility, acceptability and potential for impact of FOotpaths foR Adolescent MAternal Mental HeAlth (FOR MAMA), a co-designed intervention for pregnant adolescents in Malawi. We used a mixed-methods interventional pre-post cohort design. We recruited pregnant adolescents from a rural health centre in Zomba district, Malawi, all of whom were offered a five-session psychosocial intervention delivered by community healthcare workers. Quantitative feasibility indicators related to participant enrolment, session attendance and intervention completion. Feasibility of intervention delivery was explored using in-depth semi-structured interviews with healthcare workers. Acceptability was investigated through in-depth semi-structured interviews with intervention participants and a service user satisfaction questionnaire. Intervention outcomes were assessed using standardised measures of common mental disorders, financial distress and poor mental health and perceived social support. 19 adolescents aged 15–19 years (mean=17.21, SD=1.18) started the intervention, with 18 (94.7%) completing the programme. Significant improvements (*p*<0.05) were reported across all outcome measures, with moderate to high pre-post effect sizes. Intervention participants reported high levels of service satisfaction, although healthcare workers (*n* = 6) reported that some feasibility challenges emerged during recruitment and delivery. The *FOR MAMA* intervention proved to be an acceptable and feasible psychosocial intervention for pregnant adolescents in Malawi.

## Impact statement

Common mental health problems, particularly depressive and anxiety disorders, are among the top five causes of disability-adjusted life years (DALYs) among adolescent girls aged 15–19 globally. These conditions are further exacerbated for adolescents in the perinatal period (especially in Low- and Middle-Income Countries [LMICs]) as they are likely to experience adverse events such as stigma, lack of social support, school dropout, low self-esteem and poverty. The importance of promoting mental well-being among adolescents in the perinatal period has been emphasised as this has implications on the mental health, physical health and social outcomes of adolescents and their children. However, interventions addressing perinatal mental health problems among adolescents in LMICs, particularly in Sub-Saharan Africa remain scarce. This study is part of a larger programme that aimed to develop a contextually relevant psychosocial intervention to address perinatal common mental health problems among adolescents in Malawi. The current study aimed to evaluate the feasibility, acceptability and potential for impact of FOotpaths foR Adolescent MAternal Mental HeAlth (FOR MAMA), a co-designed psychosocial intervention for pregnant adolescents in Malawi. Significant results were found on all outcome measures, with moderate to high effect sizes. Additionally, the intervention successfully utilised task-sharing where community healthcare workers delivered the intervention to participating adolescents at both the clinic and community levels. Considering the scarcity of mental health specialists in Malawi, the task-sharing aspect is important as it can help ensure that the intervention is scalable and accessible to most adolescents. A randomised controlled trial is warranted to further evaluate the effectiveness, scalability and cost-effectiveness of the FOR MAMA intervention.

## Introduction

Adolescence is a period of elevated risk for mental health problems, especially among the subgroup of adolescents who experience pregnancy and childbirth. Some of the highest rates of adolescent pregnancies are found in Malawi, which is a country in Southeastern Africa with a population of approximately 20 million. The overall pooled prevalence of perinatal depression (i.e., during pregnancy and 12 months postpartum; Gaynes et al., 2005) in Malawi is 18.9% (Chorwe-Sungani et al., 2022), with even higher rates (up to 43.5%) among adolescents based on local surveys of postpartum depression (Tembo et al., 2023).

Although adolescent pregnancy is relatively more common in LMICs compared with high-income countries, there is scarce literature on adolescent-focused perinatal mental health interventions (Palfreyman and Gazeley, 2022). Recent systematic reviews have found small- to moderate-sized effects of psychosocial interventions targeting perinatal common mental health problems (including depression and anxiety) among adolescents (Laurenzi et al., 2020; Mhango et al., 2024a). However, evidence from LMICs is limited. Furthermore, maternal mental health research has mostly focused on postpartum depression. However, antenatal anxiety is equally prevalent (Dennis et al., 2017) and often comorbid with antenatal depression (Falah-Hassani et al., 2017), yet rarely targeted as a focal outcome. This is an important gap, considering that perinatal depression and anxiety are associated with a host of negative outcomes including preterm birth and low birth weight in babies (Siegel and Brandon, 2014; Dinwiddie et al., 2018), delayed breastfeeding initiation or early breastfeeding cessation (Field, 2018) and poor adjustment to motherhood (Oladeji et al., 2019). Moreover, recent systematic reviews on interventions for common mental health problems in the perinatal period (Waqas et al., 2022; Mhango et al., 2024a) have shown that psychosocial interventions delivered in the antenatal period can prevent both antenatal and postpartum depression and anxiety, and improve help-seeking and psychosocial functioning.

The FOR MAMA intervention was born out of the need for a contextually and age-appropriate psychosocial intervention for pregnant adolescents in Malawi, and with potential applications in other low-resource global settings. The intervention was developed through qualitative formative research followed by iterative co-design workshops (Mhango et al., 2023, 2024b), which highlighted stakeholders’ priorities and preferences for a scalable intervention for common mental health problems among perinatal adolescents in Malawi. The resulting intervention plan consisted of: (i) psychoeducation on causes, symptoms and management of common mental problems; (ii) emotion-focused strategies (relaxation techniques, anger management skills, behavioural activation); and (iii) problem-focused coping (problem-solving skills, financial literacy and interpersonal skills). The intervention was intended for delivery by community health workers using verbal instruction, skills modelling and printed booklets using both group and individual sessions.

The current study aimed to assess the acceptability, feasibility and potential for impact of FOR MAMA provided to pregnant adolescents in Malawi as a selective intervention (Compton and Shim, 2020) to prevent common mental health problems. The specific research questions were:

1. What is the acceptability of the intervention with respect to the adolescents’ user satisfaction scores and qualitative feedback from adolescents?
2. To what extent is the FOR MAMA intervention feasible considering participant enrolment, session attendance, intervention completion and qualitative feedback from healthcare workers (guides)?
3. To what extent did the intervention affect the intended outcomes considering effect sizes and confidence intervals (CIs)?

## Methods

### Study design and setting

We conducted a mixed-methods interventional pre-post cohort study from May to August, 2023. The study was conducted at Naisi health centre, a primary rural health care unit in Zomba district, Malawi. Although developmental qualitative research (Mhango et al., 2023, 2024a) was conducted at Matawale and Naisi health centres, representing urban and rural areas respectively, this formative work did not reveal any differences in contextual risk and protective factors and service needs of perinatal adolescents. Moreover, according to the most recent Demographic and Health Survey, teen pregnancies are relatively higher in rural Zomba as compared to urban areas in the same district (National Statistical Office [Malawi] and ICF, 2017).

### Participants

The Malawi Demographic and Health Survey defines teenage pregnancy as the percentage of women between the ages of 15 and 19 who are either pregnant with or have given birth to their first child – only one percent of women reported to have given birth before age 15 (National Statistical Office [Malawi] and ICF, 2017). We recruited 19 adolescents who met the following criteria:

1. were aged 15–19 years old;
2. were in the first or second trimester of pregnancy. This was recommended by healthcare workers during co-design workshops because adolescents are prone to preterm labour, hence the third trimester is considered high risk (Marvin-Dowle et al., 2018; Perez et al., 2020);
3. could read and write in Chichewa language;
4. provided consent (if aged ≥18 or considered as an emancipated minor) or assent and parental consent (if aged <18).

We excluded participants who were:

1. unable to understand and engage with the intervention material due to their inability to read and write in Chichewa;
2. at elevated risk of suicide based on whether they responded “yes” to Question 17 (In the last 30 days, has the thought of ending your life been on your mind?) on the Self Reporting Questionnaire (Beusenberg et al., 1994).

### Recruitment and consent procedures

We approached consecutive adolescent attendees at the antenatal clinic of the above-mentioned rural health centre over a three-week period. Healthcare workers provided basic information about the study and assessed those who indicated interest for eligibility. Those who were deemed eligible were referred to the lead researcher (WM) who provided further information about the study via telephone. A written information sheet was also provided to the participants in printed form. Procedures for obtaining consent were followed in line with the framework for guidelines of research in the social sciences and humanities in Malawi (National Commission for Science and Technology [Malawi], 2011). Interested adolescents aged 18 years and above, and those that were considered emancipated minors (aged below 18 but married or heading a household) provided either verbal or written consent to be enrolled into the study. An audio recording was obtained for verbal consent. For prospective participants aged under 18 years who were unable to provide consent directly, verbal or written assent was obtained in addition to verbal or written consent from a parent or guardian.

### Intervention

#### Content

The *FOR MAMA* intervention is grounded in the stress-coping theory (Lazarus and Folkman, 1984), and it aimed to equip pregnant adolescents with both problem-focused and emotion-focused coping strategies in order to prevent common mental health problems during the antenatal period and beyond. It was organised into five sessions (two delivered in groups with up to 12 participants in each group, three in 1:1 formats) delivered over five weeks (see Table 1). The goal of the introductory group session was to orient participants to the intervention, build rapport and complete a goal-setting activity (“Onboarding”). This was followed by three individual sessions spaced at weekly intervals, which respectively focused on emotion-focused coping strategies (“Managing emotions”), problem-focused coping strategies (“Handling daily problems”) and building interpersonal relationships (“Building relationships”). The concluding session (“Keeping well”) was delivered in a group format and focused on discussing potential future stressors and mitigating strategies. The group sessions lasted about an hour, while the individual sessions lasted up to 45 minutes.

**Table 1.** Intervention structure, content and delivery

Session	Content	Materials
Session 1 (group): onboarding	This session aimed to: (a) provide psychoeducation on causes, symptoms and prevention/treatment of common mental disorders; (b) provide an overview of the programme (i.e., explain the aims of the programme and how the programme works to prevent common mental disorders among pregnant adolescents); (c) introduce a case example to be used throughout the intervention; (d) identify and prioritise participants’ target problems through a goal–setting activity; (e) introduce managing emotions (1:1 session) by modelling relaxation techniques, anger management techniques and teaching behavioural activation	(a) The FOR MAMA handbook (b) Managing Emotions booklet
Session 2 (individual)	In this session, the guides: (a) reviewed activities from the managing emotions booklet; (b) taught problem–solving skills using a three–step process built around the acronym POD (REF) –– (i) **P**roblem identification which involves identifying one or more problems that are causing distress for the adolescent; (ii) **O**ption generation which involves identifying ways of addressing the different challenges identified and choosing the solution which seems best after weighing pros and cons; and (iii) **D**o it which involves implementing the chosen solution and evaluating the outcome; (c) taught financial literacy skills which included budgeting and saving.	(a) Managing emotions booklet (b) Handling daily problems booklet
Session 3 (individual):	During this session, the guides: (a) reviewed activities from the Handling daily problems booklet; (b) taught interpersonal skills and assertive communication using the booklet on Building relationships.	(a) Handling daily problems booklet (b) Building relationships booklet
Session 4 (individual):	In this session, the guides: (a) reviewed activities from the building relationships booklet; and (b) reviewed activities or clarified concepts from any of the booklets if the participants requested.	All booklets
Session 5 (group):	In this session, the guides: (a) led the adolescents in a group discussion of potential future stressors and the strategies that can be used to manage them; (b) reviewed participants goals; (c) had a closing ceremony in which they shared a meal with the adolescents.	All booklets Flip charts Sticky notes

#### Location and format

Participants were divided into two groups depending on the time they were recruited into the programme. One group that started earlier comprised eight participants and another group comprised eleven participants. Each group of participants attended two group sessions that were clinic-based and three 1:1 community-based follow-up sessions. All sessions were delivered on weekends. To ensure privacy and avoid distractions that participants would have potentially faced in a home-setting, the follow-up 1:1 sessions were held at three central convenient places within the community (e.g., schools or community health posts). Participants were divided into three clusters according to the proximity of their villages to these central places. For instance, if participants were allocated to cluster A, it meant those participants would converge at the same location within the community and each participant would have a 1:1 session with a guide in a space that was considered private by both the guide and the participant. In conjunction with the clinic- and community-based sessions, participants received four printed psychoeducational booklets in the Chichewa language (one booklet each week starting with session 1), which were used during the sessions and were intended to act as supporting material. The booklets consisted of written and illustrated material on psychoeducation on causes, symptoms, prevention and management of common mental health problems (booklet 1: The FOR MAMA handbook); emotion-focused coping strategies which included behavioural activation, relaxation techniques and anger management skills (booklet 2: Managing emotions); problem-focused coping strategies which included problem-solving techniques and financial literacy skills (booklet 3: Handling daily problems); and interpersonal and communication skills (booklet 4: Building relationships). Each booklet also included activities that participants were expected to complete at their own free time, but before the next session to demonstrate their understanding of the concepts or skills. On-demand telephone guidance was also available where participants needed clarification on how to engage with the printed material.

#### Providers

The intervention was delivered by six community healthcare workers (Health Surveillance Assistants [HSAs] and community nurses), who were referred to as “guides”, with at least one year of experience in providing maternal care to adolescents. The role of the guides was to help adolescents learn more about their mental health and how to respond to stressful things that might happen during pregnancy and beyond (postpartum) using a psychoeducational approach to the skills that were taught. This was done through verbal instruction and skills modelling during the sessions. Although the group sessions could only be run by one guide at a time, all the guides were present during these sessions to ensure continuity between the group sessions and the 1:1 sessions. Other guides supported the main facilitator by answering any questions from the participants or offering alternative explanations where necessary. The guides took turns in facilitating the group sessions. The guides were remunerated for their time at the rate of £20 per day regardless of the number of sessions they conducted on that day.

#### Training and supervision

The guides received an 8-hour initial training delivered via Zoom over two days. The training was provided by a master’s level psychologist who was also the lead intervention developer (WM) using didactic lectures and roleplay. The training covered non-specific therapeutic elements (i.e., skills that are universal to the therapy experience and impact the therapeutic outcomes) and specific elements of the intervention (i.e., elements grounded in specific psychological theories and mechanisms; Singla et al., 2017). Supplementary “just-in-time training” (McQuillin et al., 2019) was provided weekly, focusing on the forthcoming weekly topic. A printed intervention manual was made available in the Chichewa language (as per the guides’ preference). Supervision, lasting about an hour, comprised weekly group meetings moderated by WM. In each meeting, the guides discussed their experiences and received feedback from their peers and the supervisor. An opportunity for a 1:1 supervision meeting whenever deemed necessary by the supervisor or the guides was also provided.

### Measures and data collection procedures

The feasibility of the intervention was assessed using data on session attendance and intervention completion. Feasibility of research procedures was assessed through logged data on proportions of eligible/ineligible participants, consenting/assenting participants (with reasons for not consenting/assenting), and completion of outcome assessments (with reasons for not completing assessments).

Intervention outcomes were assessed via telephone by WM at baseline and post-intervention follow-up (i.e., at six weeks, counting the onboarding session as week 1) using Chichewa versions of standardised self-report measures of common mental health problems (Self Reporting Questionnaire [SRQ; Beusenberg et al., 1994]); perceived social support (Multidimensional Scale of Perceived Social Support [MSPSS; Zimet et al., 1988); and financial distress and poor mental health (Money and Mental Health Scale [MMHS; Richardson et al., 2022). The translated SRQ and MSPSS had been previously validated in Malawi with internal consistencies of α = 0.825 and α = 0.9 respectively (Stewart et al., 2013, 2014). Additional post-intervention data were obtained using a translated version of a service user satisfaction questionnaire (Client Satisfaction Questionnaire - CSQ) that had been used in other LMIC research (Michelson et al., 2020). For this study, the MMHS and CSQ were translated into Chichewa with the help of a language expert. All participants received remuneration in appreciation for their time and as reimbursement for their transportation.

Semi-structured qualitative interviews were conducted with adolescents (*n* = 18) to assess the acceptability of the intervention’s content and delivery, providing in-depth experiential evidence to complement the service user satisfaction scores. We also interviewed intervention guides (*n* = 6) to identify enablers and barriers to delivery as well as to explore recommendations for intervention refinement. All participants were interviewed via telephone by WM within three weeks of completing the intervention. Participants who did not own mobile phones were invited to complete the interview at the health centre where a mobile phone was made available to them through a research assistant. Interviews lasted 30–45 minutes and were audio-recorded, transcribed verbatim and translated from Chichewa to English.

### Safeguarding measures

The guides were trained to look out for two main safeguarding issues during the intervention: physical and/or sexual abuse and suicide ideation. Guides were to identify these risks through observation and/or spontaneous disclosure during their contact with participants. The guides would then record any incidents of safeguarding risks and discuss the immediate safeguarding action with the lead researcher based on the existing safeguarding protocol.

If a disclosure was made, for example, if the index adolescent and/or their child was raped, faced abuse or was at risk of harm, the case would be referred to the nurse in charge of the antenatal clinic (i.e., if the disclosure happened at the health centre) who would then refer the case to the local social welfare and/or child protection officer who works hand in hand with the community Police Victim Support Unit (PSVU). The PSVU provides legal and psychosocial support to women and children who are victims/survivors of abuse, violence or exploitation. The referral would either be done by phone (through the helpline) or in person. If the disclosure happened outside the health centre, the guides would refer the case directly to the PSVU. This referral system is in line with the Community-Based Complaints Mechanisms in Malawi (The Government of Malawi, 2019) which provides guidelines on reporting violence, sexual exploitation and abuse by the victim/survivor or anyone (including researchers) who has suspicion or concern.

If a participant was at risk of suicide, they would be referred to the relevant mental health professionals or organisations within 24 hours. A phone call would be made to a relevant organisation (e.g., Youth Net and Counselling). If contact was unsuccessful at first attempt an email would be sent. If no response is received within six hours or by the end of business hours, a referral would be made to the central or psychiatric hospital. The researcher would work hand in hand with the nurse in charge or the officer in charge of the health centre to ensure that the adolescent was accompanied to the hospital/organisation and monitored for safety.

### Data analysis

Quantitative indicators of feasibility and acceptability were analysed using descriptive statistics. Psychosocial outcomes were analysed using paired samples t-tests. The results were summarised using means, standard deviations, effect size (with 95% CIs) or frequencies/proportions, as appropriate. Interview data were analysed using the Framework Analysis method (Gale et al., 2013). This involved transcription, familiarisation, coding, developing an analytical framework, indexing, charting and interpreting the data. The analysis followed both an inductive data-driven approach and a deductive approach. For interviews with adolescents, findings were organised around the constructs of the Theoretical Framework of Acceptability (Sekhon et al., 2017). Data analysis was led by the first author (WM) with regular input from the senior co-authors (DG & DM).

## Results

### Uptake and completion of the intervention

**Figure 1.**
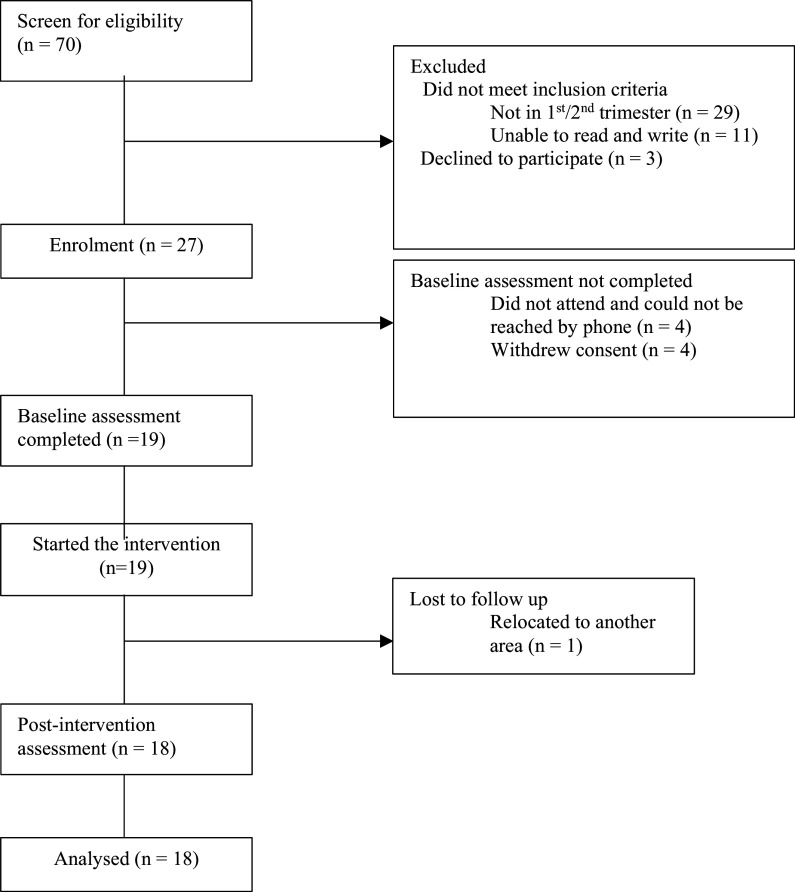
Flow diagram.

We approached 70 participants out of whom 40 did not meet the eligibility criteria due to being in their third trimester of pregnancy (n=29) or due to illiteracy (n=11). Of the 30 eligible adolescents, three (10%) declined to participate as they felt they did not have the time and could not take on the burden of participating in the programme. Twenty-seven of the eligible participants (90%) were enrolled in the study, out of whom 19 (63.3%) completed the baseline assessment. The eight participants who did not complete the assessment could not be reached by telephone (n=4) or else changed their mind and withdrew consent (n=4). Out of the 19 participants who started the intervention, 18 (94.7%) completed all five sessions while one participant could attend only three sessions due to relocating to a different area (see Figure 1).

Intervention participants were aged 15–19 years (mean = 17.21, SD = 1.18). The majority (94.7%) of the participants had dropped out of school with over half of them citing pregnancy as a reason for dropping out. Over half of the participants were married. (Table 2).

**Table 2.** Sociodemographic characteristics of intervention participants (N=19 unless otherwise specified)

Age of participants	
Mean (SD)	17.21 (1.18)
Range	15–19
Marital status, *n* (%)	
Married	13 (68.4)
Single	6 (31.6)
Gestational period, *n* (%)	
First trimester	2 (10.5)
Second trimester	17 (89.5)
Pregnancy, *n* (%)	
Planned	6 (31.6)
Unplanned	13 (68.4)
Education level, *n* (%)	
Primary	17 (89.5)
Secondary	2 (10.5)
Education status, *n* (%)	
Dropped out of school	18 (94.7)
Currently in school	1 (5.3)
Reasons for school dropout, *n* (%)^1^	
Pregnancy	13 (72.2)
Financial challenges	4 (22.2)
Personal choice	1 (5.6)
Source of income, *n* (%)	
Piece work	9 (50.0)
Business	1 (5.6)
Family members	8 (44.6)

1 *N* = 18.

### Psychosocial outcomes of the intervention

Table 3 provides data for all outcome measures at pre-test and post-test. Pre-post comparisons showed statistically significant improvement on all measures, with large effects observed on the SRQ scores (g = 0.89; 95% CI 0.35 to 1.41; p = 0.001) and MSPSS scores (g = −2.36; 95% CI −3.25 to -1.45; p < 0.001). Moderate effects were observed on MMHS scores (g = 0.76; 95% CI 0.24 to 1.26; p = 0.004).

**Table 3.** Psychosocial outcomes

	Pre-intervention	Post-intervention	Comparison		
Outcomes	Mean (SD)	Mean (SD)	Mean diff (SD)	Hedges’ g (95% CI)	*p*
CMD symptoms (SRQ)	8.56 (3.57)	6.28 (3.32)	2.28 (2.44)	0.89 (0.35 to 1.41)	0.001
Social support (MSPSS)	25.44 (6.08)	34.06 (6.36)	−8.61 (3.48)	−2.36 (–3.25 to –1.45)	< 0.001
Financial distress and poor mental health (MMHS)	15.50 (7.65)	12.17 (6.21)	3.33 (4.20)	0.76 (0.24 to 1.26)	0.004

### Acceptability of the intervention: Interviews with FOR MAMA participants

#### User satisfaction

The mean satisfaction score was 29 (SD = 2.59; Range = 22–32) out of a maximum of 32. All participants indicated that they were ‘mostly satisfied’ or ‘very satisfied’ with the service they received, and they all reported that they would ‘definitely’ participate in the programme again.

### Perceived effectiveness

Participants highlighted the effectiveness of the different components of the intervention (see Table 4). All booklets were perceived as being effective. However, the booklet on “Building Relationships” was particularly appreciated by most adolescents as it equipped them with skills for building new relationships and repairing broken relationships. Most adolescents described experiences of losing friends and/or falling out with their family members due to the shame and stigma of teen pregnancy.

“*A lot of people don’t want to talk to us because we are pregnant at a young age. So, it was good that this book taught us how we can build relationships. Everyone wants a friend they can talk to.” (Pregnant adolescent #6, 19 years old, married)*

**Table 4.** Themes and illustrative quotes from exit interviews

*Acceptability of the intervention: Adolescent interviews*	
Affective attitude	*“I liked the programme because it had those booklets. I like reading…The stories in the booklets were interesting and easy to understand.” (Pregnant adolescent #12, 19 years old, married)* *“My heart was in it, I liked the programme, so I wouldn’t forget to read the booklet and do the activities.” (Pregnant adolescent 1, #18 years old, married)*
Perceived effectiveness	Intervention booklets/components *“The part on relaxation techniques…It helped me feel more relaxed because most of the times my body would feel very tense, especially when I am worried about something” (Pregnant adolescent #7, 17 years old, married)* *“When I was coming into the programme, I did not know how to solve problems. I would just worry. Now I have learned how to come up with solutions to different problems.” (Pregnant adolescent #16, 17 years old, unmarried)* Activities *“When you just read something without practicing, you think you have understood. But when you actually do those activities that is when you know whether you have really understood or not. If some things are hard, then you ask to understand better.” (Pregnant adolescent #6, 19 years old, married)* Providers *“If they just gave us books without being involved, I don’t think we would have understood the books very well. But before giving us the books, they would explain what the book is about, so we read them knowing what exactly the book is trying to teach us.” (Pregnant adolescent #10, 19 years old, married)* *“The healthcare workers were so friendly, they did not judge us. So, it was like going to talk to your big sister.” (Pregnant adolescent #8, 15 years old, married)* *“They were very encouraging. Apart from just reading the booklets, most of us were able to open up to them about the challenges we were facing and the things we were worried about.” (Pregnant adolescent #14, 17 years old, married)* Dosing *“I feel meeting once a week was okay. If the time is longer, a lot of things might happen and one might end up forgetting about the programme. Meeting every week gave me something to look forward to.” (Pregnant adolescent #2, 16 years old, married)* Format *“I would still prefer both the group and individual sessions. In a group I felt like I was not alone and we would share experiences. In individual session, I was free to talk about my own issues.” (Pregnant adolescent #17, 17 years old, married)* Location *“I preferred attending the intervention in the community because I did not have to walk a long distance. But I did not mind going to clinic sometimes because we would meet as a larger group. The clinic was also a central place for everyone.” (Pregnant adolescent #5, 17 years old, unmarried)* *“Both locations had their own advantages. When we are meeting at clinic, we would walk for a long distance so it was like exercise. But, you don’t want to be walking all the time. So, meeting in the community helped us to rest from walking.” (Pregnant adolescent #11, 16 years old, unmarried)*
Self–efficacy	*“I was okay doing everything by myself because I was confident that I knew what I was doing…Because I went to school. I know how to read and write…You do not write an exam with anyone, you do it by yourself. That’s why I did it by myself”. (Pregnant adolescent #1, 18 years old, unmarried)* *“My relatives were assisting me…When we were given the tasks, I would ask my relatives to help me complete them because I was not sure if I was doing them correctly.” (Pregnant adolescent 9, #17 years old, married)*
Intervention coherence	*“Because I do not have worries, I am able to help around with the house chores, especially now that I know how to plan my day by writing down what I want to do in the morning, afternoon and evening. I am happier so I also relate with my family well. They are also happy.” (Pregnant adolescent #13, 18 years old, married)* *“I’d say it’s a program that helps with mental health…The books had different lessons that were helping us have good mental health. If you practice those relaxation techniques your mind is also free. If you follow those steps on problem solving, you can come up with solutions to any problem. In the end, you will be happier. You will not have worries.” (Pregnant adolescent #5, 17 years old, unmarried)*
Burden and opportunity cost	*“The intervention did not interfere with my activities because it was brief… I was reading the books after completing my chores.” (Pregnant adolescent #10, 19 years old, married)* *“I did not mind spending time to complete the activities…It helped me focus my time on doing that instead of just sleeping. I found something to do…I was enjoying the programme and I wanted to see the end line of the program.” (Pregnant adolescent #7, 17 years old, married)*
*Feasibility of intervention delivery: healthcare worker interviews*	
*Facilitators*	
Perceived benefits	*“For me, I value the experience I have gained. I think it is important especially considering that we handle these young mothers on a weekly basis. In addition, a lot of people trust health care workers as compared to lay persons. They would consider the thing is shallow. But when a health care worker is involved, it carries more weight. They feel it is important and they are more likely to come.” (Guide #3)*
Training and supervision	*“The training of the providers helped ensure that there was consistency in the delivery of the programme.” (Guide #2)* *“To be honest, on the first day I found it a bit difficult. You know, it is something I have never done before so I was not sure if I was going in the right direction. But after the supervision meeting for that first meeting and receiving feedback on what I could do better, I felt more confident.* *Things became more straightforward as we went on.” (Guide #3)*
Use of booklets	*“I think the use of the booklets was helpful. For the adolescents, it was easy for them because they all knew how to read and write. If they forget something, they would go back to the booklet and read it again.” (Guide #5)*
Sense of ownership	*“It felt like our thing, our baby. We wanted to see it from start to finish.” (Guide #6)*
Remuneration	*“The programme was important. But being paid made me feel like my time was valued. I did not mind putting in the extra work.” (Guide #2)*
Scheduling and flexibility of sessions	Scheduling *“Meeting the clients weekly kept them busy unlike having a longer duration, one might get relaxed because they think they have a lot of time, so they might forget to complete the activities and practice.” (Guide #3)* *“It is even better than the sessions were held on weekends because both we the health workers and the adolescents were free during weekends. Weekdays are usually busy.” (Guide #1)* Flexibility of sessions *“The length of the sessions was very flexible. If the participant is fast in grasping concepts, the sessions would be short. So, we would allocate the extra time to those who were slower. It worked perfectly.” (Guide #4)*
*Barriers*	
Lateness of participants	*“Some participants would come late so we had to wait for them, especially for the group sessions. Most of them did not even have phones where we would contact them to ask if they were going to come.” (Guide #2)*
Community attitudes towards the programme	*“Some community members were also spreading false information by telling the girls that this was a satanic thing and that we were going to get the blood of their babies. So some would feel discouraged.” (Guide #3)*
*Suggestions for improvements*	
Additional sessions	*“The sessions were enough. But I think we should have one or two extra sessions in case anything happens, for instance funerals, and participants are unable to show up for sessions. It did not happen during this programme. But it would be good to have it as a contingency plan.” (Guide #1)*
Raising community awareness	*“We need to raise awareness in the communities. If the programme will continue, we can use the girls who participated in this programme to give testimonials that the programme is not harmful and maybe mention how they benefitted from the programme. Otherwise, people will keep spreading false information.” (Guide #2)*
Inclusion of those who cannot read and write	*“For those we would have to read booklets to them and then complete the activities with them. They will be responding to the questions and you as the guide will now write down those responses. If they have someone at home who can read then even better.” (Guide #4)*

#### Affective attitude

This theme captured adolescents’ feelings about FOR MAMA. Adolescents stated that the best things about the programme were opportunities for them to interact with their peers; engage in purposeful activities instead of just staying home with nothing to do; and have access to interesting, easy-to-read booklets.

*“I enjoyed taking part in the programme. I wish the programme was longer because it was very interesting, especially meeting with other girls.”*

*(Pregnant adolescent #15, 16 years old, unmarried)*

“*I liked the booklets because I found something to read. I learnt how to deal with different things and also feel better, but I was also reading them to pass time. The stories were interesting and very clear.”*

*(Pregnant adolescent #3, 17 years old, unmarried)*

#### Self-efficacy

Most adolescents reported that they were confident to engage with the intervention material independently before the follow-up meetings. A smaller number of interviewees explained that they sought help from family members when they were unsure of what to do or how to complete the activities. None of the adolescents sought additional help from the guides using the phone call option that was available for them in between sessions.

“*I was very confident of what I was doing because I had my mother to discuss with. The health workers were also there to check if we had done the activities correctly. For me, the health worker who was guiding me said I did most of my work correctly. So that also added to my confidence, and it just showed that I had understood what I read.*”

*(Pregnant adolescent #14, 17 years old, married)*

#### Intervention coherence

Participants demonstrated a good understanding of the purpose of the FOR MAMA programme, making direct links between skill development and reduced psychological distress. Adolescents readily provided examples of applying newly learned skills to real-life challenges, such as using relaxation techniques when they feel anxious, using problem-solving skills to identify ways of finding money and applying the knowledge from the financial literacy module to make a plan on how to save or come up with a budget in preparation for the baby they were expecting.

“*When we were setting goals, one of my goals was to find money to buy things for my baby. So, when we learned about problem-solving, one of the solutions I came up with was to do manual labour. Then I used what we learned about coming up with a daily plan of activities to write down when I can do the manual labour.*”

*(Pregnant adolescent #4, 17 years old, married)*

Moreover, the mixed intervention format (group plus individual sessions) was valued by most participants. Adolescents appreciated the opportunities for peer support in the group sessions, which they saw as an important complement to private 1:1 sessions with kind, friendly and non-judgmental guides.

*“In a group, I felt like I was not alone and we would share experiences. In individual session, I was free to talk to the guide about my own issues.”*

*(Pregnant adolescent #11, 16 years old, unmarried)*

#### Burden and opportunity cost

Adolescents reported that the programme did not interfere with other activities because the brief booklets did not require a lot of time to read and in-person sessions could be accommodated within their normal weekly schedule. Indeed, some adolescents mentioned favourably that the programme kept them occupied since they had dropped out of school and did not have anything to do.

*“I was learning things that I felt were useful for me and I was also happier. There were other times when I had nothing to do, so reading the booklets kept me occupied instead of just worrying.”*

*(Pregnant adolescent #15, 16 years old, unmarried)*

Although some adolescents complained about the distance to the clinic when attending the group sessions, others felt that walking to the clinic provided a helpful impetus for them to exercise. Moreover, the clinic provided a common meeting point that allowed them to interact with peers.

*“The clinic is a bit far, but I did not mind going there for some sessions because it gave me the opportunity to exercise.”*

*(Pregnant adolescent #2, 16 years old, married)*

### Feasibility of intervention delivery: Interviews with FOR MAMA guides

#### Facilitators

The guides mentioned several factors that facilitated the delivery of FOR MAMA.

##### Perceived benefits

The guides felt that they also benefited from the programme as they had gained new knowledge and skills that were relevant to their normal area of practice. Although they had prior healthcare experience with pregnant adolescents, the FOR MAMA programme afforded new learning opportunities through which they understood the unique psychological challenges faced by adolescents, particularly when pregnant.

“*This programme helped us update our knowledge of mental health and it also taught us the approaches we can use when we are dealing with young girls because they are more at risk of mental health problems.*”

*(Guide #1)*

##### Training and supervision

Guides appreciated access to training and supervision in equipping them with the necessary knowledge and skills on causes and symptoms of mental health problems and how to equip adolescents with various coping strategies. Some healthcare workers felt that supervision increased their confidence in delivering the programme as they received feedback on the sessions that they delivered as well as suggestions on how they could improve delivery for the subsequent sessions. All guides felt that the training and supervision that they received were adequate. However, some reported that they would have preferred face-to-face training to prevent online fatigue.

*“I was confident because we had the training on how to go about delivering the content. And every week before meeting the clients, [the supervisor] was guiding us again on what was supposed to be done in the next session. That gave us the confidence to deliver the information to our clients in the manner that it was supposed to be done.”*

*(Guide #3)*

##### Use of booklets

According to the guides, the booklets ensured that the adolescents were receiving the same message irrespective of who was delivering the session. Consistent with the adolescent interviews, the guides commented favourably on the brief and engaging booklet formats.

*“Without the booklets, it would have been easy for us to be teaching those girls different things, but with the booklets and manuals, we were all doing or saying the same things.”*

*(Guide #6)*

##### Sense of ownership

All healthcare workers involved as FOR MAMA guides had taken part in earlier co-design workshops (conducted before the start of the current study). Following from this formative experience, several guides described a sense of ownership and explained that they were motivated to deliver the intervention to ensure its success. Participating in the co-design workshops had also provided guides with a clear understanding about the purpose of the programme and appreciation for its potential strengths.

*“We were involved in developing the content that we needed to deliver to the clients, that was helpful, it gave us a sense of ownership.”*

*(Guide #2)*

##### Remuneration

Some guides reported that they were more willing to deliver the programme because they were getting remunerated. This made them feel that their time and efforts were being appreciated and that they were not just taking on extra work for free.

*“I am not going to lie, there was the monetary incentive that was motivating us, it was not just voluntary.”*

*(Guide #4)*

##### Scheduling and flexibility of sessions

Guides noted that flexible timings of sessions allowed sufficient time to develop personalised responses to adolescents’ questions and concerns.

*“Some would grasp the things within a short period. For those who could grasp the concepts quickly, it wasn’t time consuming. But some were still taking time but we accommodated them. So we didn’t have a fixed time for the sessions. It was dependent on the client.”*

*(Guide #6)*

The intervention providers also appreciated the fact that both the group and individual sessions were delivered on weekends as the clinic was less busy and they were less likely to be on duty for their main role. Otherwise, they easily swapped days with other healthcare workers who were not participating in FOR MAMA.

*“If I was on duty at work, I would just swap with a colleague. Someone will work during my shift and I will work during theirs. The good thing is that weekends are usually not busy because we do not run routine clinics.”*

*(Guide #5)*

#### Barriers

##### Lateness of participants

Guides noted the difficulties of running group sessions when one or more participants attended late and missed out on parts of the group discussion, as well as disrupting the flow for other attendees. Most of the adolescents did not own mobile phones making it difficult for the guides to communicate with them to find out whether they would show up for the sessions.

*“The main challenge [with group sessions] was that we had to wait for all or most of the participants to come. Some would come on time, others would be late and find that we had already started.”*

*(Guide #1)*

##### Community attitudes towards the programme

It was noted that some community members (i.e., family members, friends and neighbours of potential participants) had provided inaccurate and unhelpful warnings to study participants. This seemed to stem from previous research activities in the community, which had involved “drawing blood from babies.” This concern about physically intrusive procedures, with the potential to harm unborn babies, appeared to be the basis for several participants withdrawing consent before starting the intervention. The guides explained that other adolescents had initiated the intervention despite similar misgivings about taking part and had considered dropping out. Consequently, guides personally visited concerned family members to offer reassurance about the nature of the intervention and associated research activities.

*“At the start of the programme, some girls wanted to discontinue because people were scaring them that we will take their blood and their babies will die. We had to explain to some of their family members that there wasn’t going to be anything like that. Some stayed, but others still did not want to continue.”*

*(Guide #5)*

#### Suggestions for improvements

##### Raising community awareness

All guides suggested that it may be beneficial to raise awareness about the programme in the target communities before the start of the programme to dispel any misconceptions about the programme. This can be done by engaging community leaders and the wider community in meetings where they can be sensitised on the aims, procedures and potential benefits and risks of the programme.

*“Before starting the programme, we need to engage the community to explain what the programme is about. That way, everyone will be on board and people will be less resistant. It can be done easily with the help of chiefs and other community leaders.”*

*(Guide #6)*

##### Inclusion of those who cannot read and write

It was recommended that FOR MAMA should be inclusive of adolescents who were unable to read and write, as these young people were equally at risk of developing common mental health problems compared with literate peers. Some guides suggested that they would be willing to read the booklet to the adolescents. In addition, during the recruitment process, adolescents could be asked to identify family members who could read and write to assist them.

*“I think one option would be to include them if they have someone who is willing to help them when they go home. That means they would have to come with that person to the sessions so that that person can be in a better position to help them when they go home. Because despite the fact that they cannot read and write, they can still face some challenges that can affect their mental health. So we can either include a guardian or spouse.”*

*(Guide #2)*

##### Addition of extra sessions

Guides suggested that one or two extra optional 1:1 sessions could be added to the programme. This would cater to those participants who were unable to complete the intervention activities in time due to life circumstances such as illness, funeral (loss of a family or community member) or family stress.

“*I think we should have had an extra week after the last booklet but before the final session where we would just go through all the booklets so that those that are a bit behind can catch up. This session can even be optional, but it would help the ones who are behind.*”

(Guide #5)

Additional illustrative quotes on the acceptability and feasibility of the intervention have been presented in Table 4.

## Discussion

To the best of our knowledge, this is the first study to evaluate the feasibility, acceptability and potential effects of a co-designed mental health intervention for pregnant adolescents in Malawi. The quantitative findings indicate that the intervention could potentially reduce symptoms of psychological distress and improve perceived social support and perceived financial well-being. We found large effect sizes on measures of perinatal psychological distress (SRQ) and perceived social support (MSPSS). These effect sizes are larger than the effect sizes reported in most adolescent maternal mental health interventions (Laurenzi et al., 2020; Osborn et al., 2020). However, similar large effect sizes have been reported for a study of a psychosocial intervention for the prevention of postpartum depression in adolescent mothers (Sangsawang et al., 2021). Moreover, moderate effect sizes were observed on the financial well-being measure (MMHS). Both quantitative and qualitative evidence suggest high levels of acceptability and overall service satisfaction. These results support findings that psychosocial interventions that utilise task-sharing are effective in preventing perinatal mental health problems among women in LMICs (Prina et al., 2023).

The study had a high consent rate (90%) among those who met the eligibility criteria. Furthermore, the intervention had a high completion rate (94.7%). It was reported that regular meetings with the healthcare workers kept the adolescents motivated to continue engaging with the intervention. This is in line with studies that suggest that purely self-directed interventions have lower engagement rates than interventions with human support, even if the amount of guidance is relatively slight (Parikh et al., 2019). In addition, conducting follow-up sessions in the participants’ communities mitigated the distance barrier, thereby making it easier for participants to attend the intervention sessions (Tomlinson et al., 2020). Moreover, the use of co-design methods during earlier stages of intervention development may have increased the overall acceptability of the programme (Yardley et al., 2015), as suggested by the healthcare workers during the interviews.

A recent systematic review found mixed results regarding the efficacy of interventions based on their duration (Mhango et al., 2024a). However, the current study found that a brief intervention (i.e., five lessons delivered over five weeks) was feasible, acceptable and effective. Efficacy has also been reported in adolescent mental health interventions (Michelson et al., 2020; Gonsalves et al., 2021) and maternal mental health interventions (Chibanda et al., 2014) of similar or shorter duration and dosing that were conducted in LMICs. Although some research has shown that longer interventions can help participants build trusting relationships with the intervention providers (Barry et al., 2013), all participants in this study reported that they valued the positive relationships they developed with the guides as it was seen to increase self-efficacy. Adolescents also appreciated the positive qualities (non-specific therapeutic aspects) of the guides as this helped them to open up. This adds to the very large body of research showing that a strong therapeutic alliance is vital to engagement and effectiveness of youth mental health interventions (Labouliere et al., 2017; Persson et al., 2017).

Although stigma associated with teen pregnancy and mental health problems has been found to be a barrier to service access (Mutahi et al., 2022), no cases of mental health stigma were reported in this study. However, some participants considered withdrawing from the intervention due to some community members spreading misconceptions about the study. Future studies could benefit from raising awareness about the programme in the communities prior to beginning the programme. This might further enhance the overall acceptability of the programme in the wider community and increase enrolment rates. A study on community and healthcare provider perspectives on the ethicality and acceptability of maternal mental health research in Malawi (Ndambo et al., 2023) found that community members were more open to maternal mental health research if the researchers engaged the community gatekeepers (i.e., chiefs/traditional leaders, religious leaders or political leaders) before recruiting participants. These community gatekeepers would then mobilise the community members and even recommend the best approach to working in those communities, thereby helping the researchers build community trust.

The opportunity to build social networks through interactions with other adolescents was valued by most adolescents. Some reported that interacting with peers gave them a sense of belonging as they usually faced social isolation due to the stigma associated with teen pregnancy. This echoes other intervention research in which peer group formats have been identified as an important source of emotional and informational support for adolescents in the perinatal period (Fang et al., 2022). Nevertheless, adolescents still valued the individual sessions, which enabled them to discuss confidential issues without the fear of a group member disclosing personal information outside the group.

The module on financial literacy was appreciated by most participants in the study as it equipped adolescents with specific knowledge and skills on how they can manage their finances to achieve their financial goals, such as buying materials in preparation for childbirth. Although previous research in LMICs has identified financial instability as a major challenge that may potentially lead to poor mental health among adolescents in the perinatal period (Taylor Salisbury et al., 2021; Palfreyman and Gazeley, 2022; Mhango et al., 2023), mental health interventions for adolescents have hardly focused on specifically addressing this challenge. Participants in our study highlighted that, although the problem-solving module equipped them with ways in which they can address various challenges (e.g., coming up with ways to generate income) the financial literacy module equipped them with deeper knowledge on how they can effectively manage the finances. This implies that interventions that incorporate elements that target specific common life challenges among adolescents may be beneficial in improving common mental health problems among adolescents.

Although participants had the option of contacting the guides via telephone between sessions, none of the participants took up this option. This could be because participants were confident in completing the activities by themselves or they had other people (friends or family members) helping them, as reported in this study by some adolescents. Moreover, adolescents met the guides weekly, hence they may not have felt the need to use the phone call option in between sessions. It might also be due to the fact that most adolescents did not possess mobile phones. Despite the widespread enthusiasm about digital or mobile health (mHealth) interventions to improve service access for maternal health (Colaci et al., 2016) and mental health among adolescents in settings with limited human resources (Madonsela et al., 2023) implementation in this study was not possible as most adolescents do not own mobile phones or internet-enabled devices. Hence, the intervention was fully delivered in person.

It is important to note that this was a psychoeducational programme in that the focus was to teach adolescents various problem-focused and emotion-focused coping skills through verbal instruction, skills modelling during the sessions as well as didactic information as written down in the booklets. The intervention incorporated some insights from a mixed-methods systematic review that we had conducted on psychoeducation as an ‘active ingredient’ for perinatal depression and anxiety in youth (Mhango et al., 2024a). Our experience in this study was that adolescents particularly appreciated the opportunity to engage with their peers during group sessions as well as positive interactions with the guides. The intervention was also deemed to be beneficial in equipping the adolescents with skills that helped them cope with real life challenges. These aspects were also endorsed by our earlier lived experience panel (Mhango et al., 2024a).

### Strengths and limitations

A key strength of this study was the use of both quantitative and qualitative methods. This enabled us to generate rich descriptions of the acceptability, feasibility and effects of the intervention. However, the study has some limitations. The current study was conducted at one of the sites where the formative work was carried out. However, for future studies with involvement of multiple sites, it will be beneficial to conduct a pilot feasibility study to assess the sites’ readiness for implementation and refine the intervention as needed. We used a relatively small sample. This limits the conclusions that can be drawn from this study. In addition, we did not use a control group. Therefore, the improved outcomes might have been due to other factors (e.g., spontaneous remission) aside from the specific effects of the interventions. Moreover, half of the participants were married. Previous research (Munthali and Kok, 2016; Mhango et al., 2023) found that marriage could have a protective effect on mental health among pregnant adolescents as it provided a source of social support and reduced pregnancy-related stigma, that is, those who were married were seen more favourably by society as pregnancy out of wedlock is often frowned upon. Furthermore, follow-up assessments were only conducted at six weeks with no further follow-up. Therefore, it is difficult to ascertain whether the effects of the intervention were maintained and whether participants fully internalised and mastered the skills.

We used self-report measures. Therefore, it is possible that there was a social desirability bias (Fisher and Katz, 2000) where participants may have underreported or exaggerated their experiences to project a positive image of themselves. The MMHS and CSQ have not been validated in Malawi, hence the psychometric properties of these instruments in this context are unknown. Telephone interviews were utilised to collect data. Much as this is a common practice for quantitative interviewing, it presents some limitations for qualitative interviewing such as poor rapport building that may affect the quality of data, inability to read visual cues and participant fatigue if the interviews are long (Sturges and Hanrahan, 2004; Opdenakker, 2006). In an attempt to address these issues in this study, the interviewer engaged in an informal chat with each participant to build rapport at the start of the interview process. In addition, the length of the interviews was less than one hour to prevent fatigue. A systematic review of studies on telephone interviewing in qualitative research (Novick, 2008) also found that telephone interviews allow for more anonymity, hence participants are more comfortable disclosing sensitive information. Lastly, we excluded participants in their third trimester due to the risk of preterm birth in adolescents (Marvin-Dowle et al., 2018; Perez et al., 2020). However, taking into account the brief nature of the intervention, future studies should consider including participants who are at the beginning of their third trimester.

## Conclusions

This study found that the FOR MAMA intervention was acceptable, feasible and potentially effective in improving symptoms of common mental health problems, enhancing perceived social support and reducing money worries among pregnant adolescents in Malawi. However, this was not a controlled study. Hence, a randomised controlled trial is needed to further evaluate the effectiveness of an updated version of FOR MAMA. The flexibility and simplicity of the FOR MAMA intervention provides an opportunity for scale up within Malawi and in other low-resource global settings. The FOR MAMA intervention is a low-cost intervention that can be delivered by non-specialist healthcare workers or laypersons in settings with limited mental health specialists. Furthermore, the intervention material can easily be adapted to suit the language and culture of different contexts. Moreover, the brief nature of the intervention sessions provides an opportunity for the intervention to be integrated into the primary healthcare system to increase accessibility of mental health services to pregnant adolescents, thereby addressing the treatment gap.

## Data Availability

Due to conditions of participant consent, the data from this study are not publicly available.
