# Supplementary material for: Feasibility and acceptability of FOotpaths foR adolescent MAternal mental HeAlth (FOR MAMA): A co-designed intervention for pregnant adolescents in Malawi
Source: Glob Ment Health (Camb). 2024 Oct 24;11:e97. doi: 10.1017/gmh.2024.76 (PMC11504933; doi:10.1017/gmh.2024.76)
Supplement: Peer review history

# Editor report: Recommendation: Feasibility and acceptability of FOotpaths foR adolescent MAternal mental HeAlth (FOR MAMA): A co-designed intervention for pregnant adolescents in Malawi — R0/PR2

**DOI:** 10.1017/gmh.2024.76.pr2

**Affiliations:** School of Public Health and Preventive Medicine, Monash University Faculty of Medicine Nursing and Health Sciences, Australia

No accompanying comment.

# Editor report: Decision: Feasibility and acceptability of FOotpaths foR adolescent MAternal mental HeAlth (FOR MAMA): A co-designed intervention for pregnant adolescents in Malawi — R0/PR3

**DOI:** 10.1017/gmh.2024.76.pr3

**Affiliations:** London School of Hygiene & Tropical Medicine, United Kingdom of Great Britain and Northern Ireland

No accompanying comment.

# Editor report: Recommendation: Feasibility and acceptability of FOotpaths foR adolescent MAternal mental HeAlth (FOR MAMA): A co-designed intervention for pregnant adolescents in Malawi — R1/PR5

**DOI:** 10.1017/gmh.2024.76.pr5

**Affiliations:** School of Public Health and Preventive Medicine, Monash University Faculty of Medicine Nursing and Health Sciences, Australia

No accompanying comment.

# Editor report: Decision: Feasibility and acceptability of FOotpaths foR adolescent MAternal mental HeAlth (FOR MAMA): A co-designed intervention for pregnant adolescents in Malawi — R1/PR6

**DOI:** 10.1017/gmh.2024.76.pr6

**Affiliations:** London School of Hygiene & Tropical Medicine, United Kingdom of Great Britain and Northern Ireland

No accompanying comment.
